# Adaptations in anesthesiology residency programs amid the COVID-19 pandemic: virtual approaches to applicant recruitment

**DOI:** 10.1186/s12909-021-02895-2

**Published:** 2021-08-31

**Authors:** Donaldson C. Lee, Alexander M. Kofskey, Nikhi P. Singh, Timothy W. King, Paul D. Piennette

**Affiliations:** 1 School of Medicine, University of Alabama at Birmingham, Birmingham, AL 35233 USA; 2 Department of Anesthesiology and Perioperative Medicine, University of Alabama at Birmingham, Birmingham, AL USA

**Keywords:** Residency, Application, Virtual, COVID, Social media, Open house, Recruitment

## Abstract

**Background:**

The COVID-19 pandemic significantly impacted residency recruitment in 2020, posing unique challenges for programs and applicants alike. Anesthesiology programs have adopted alternate methods of recruitment, including virtual open houses and social media, due to limiting personal contact rules implemented by AAMC. This study was undertaken to determine the frequency of virtual events hosted and social media accounts created by programs.

**Methods:**

Anesthesiology residency programs and departments were examined for social media presence on Twitter, Instagram, and Facebook. Programs’ websites and social media posts were reviewed for virtual open house opportunities. Available sub-internships were collected from the Visiting Student Application Service database. Data was collected after 2020–2021 pre-interview recruitment in October 2020.

**Results:**

Of 153 total anesthesiology residency programs, 96 (63%) had some form of social media presence. The platforms of choice for programs with social media accounts included Twitter (71, or 46%), Instagram (67, or 44%), and Facebook (47, or 31%). Forty of seventy-six residency-affiliated accounts were created after March 1, 2020; Instagram accounts (26 of 40) represented most of these. Most Anesthesiology programs (59%) offered virtual open houses for prospective applicants. Twitter (25%), Instagram (22%), and Facebook (8%) were used by programs to advertise these events.

**Conclusions:**

Social media presence of anesthesiology residency programs has grown steadily over the past decade, with exponential growth experienced in 2020. This data suggests that anesthesiology residency programs are employing new, mostly virtual, methods to reach prospective applicants during an unprecedented application cycle amidst the COVID-19 pandemic.

## Background

The COVID-19 pandemic significantly impacted the 2020–2021 resident recruitment process [1]. Historically, visiting away rotations, or sub-internships (sub-I’s), have played a role to assist residents’ matching into highly competitive specialties, often at applicants’ preferred institutions. In an Association of American Medical Colleges (AAMC) survey of 2019 medical school graduates, 42.8% of prospective anesthesia residents completed an away rotation [2]. Since the onset of the COVID-19 pandemic in the United States in early 2020, nearly all residency programs have suspended visiting away rotations and in-person interviews, following guidance from AAMC [3]. The American Society of Anesthesiologists (ASA) similarly recognized the unique difficulties that COVID-19 has placed on current residency applicants, emphasizing the need for increased virtual opportunities [4]. Without these unique experiences, students may lose valuable insight into programs where they may be interested in matching. Similarly constrained, programs may not be able to adequately assess and restrict their potential applicant pool.

Social media platforms present a mutually beneficial opportunity for residency programs to share information with prospective residents and for potential residents to evaluate residency programs. A previous survey across three anesthesia programs demonstrated that 52.8% of incoming residents considered information viewed on a prospective program’s social media accounts to impact their rank list [5]. We assessed how anesthesiology programs have employed virtual opportunities, i.e., open houses^1^ and sub-internships,^2^ and social media to showcase their program attributes and interact with prospective residents in the unprecedented 2020–2021 application cycle.

## Methods

After receiving exempt status from the UAB Institutional Review Board for Human Use, a list of all accredited anesthesiology programs was obtained from The Electronic Residency Application Service (ERAS); 153 total programs were identified. Through internet searches and investigation of each program’s website, it was determined that programs’ social media presence was exclusive to Twitter, Instagram, and Facebook. Each social media platform was examined to determine the existence of department and residency-affiliated program accounts. Residency-affiliated accounts included those run by administrative personnel, faculty, or residents and were labeled as such on their profiles; department accounts were considered as all other institution-specific anesthesiology accounts without an explicit description of being affiliated with a residency program. Accounts were reviewed for their listed creation date (or date of first post), virtual open house, and virtual sub-internship offerings. Accounts were determined to be officially associated with various anesthesiology department and residency programs by qualitative observation of the content and linkage to official residency and department websites. Open house opportunities were recorded by how many independent (non-ASA or AAMC associated), program-specific events were listed on each platform. Careful observation was performed to ensure that individual events were counted once, despite many being advertised with multiple successive posts. Program websites and an ASA-sponsored database [6, 7] were also investigated for virtual open house and virtual sub-internship opportunities. The AAMC Visiting Student Application Service (VSAS) was reviewed to identify virtual sub-internship opportunities. Data was collected from October 15, 2020, to October 31, 2020, as this time frame followed the pre-interview recruitment season, i.e., when most open houses would have already been held, and roughly coincided with the date (October 21, 2020) when programs could begin viewing applications [8].

## Results

Social media existence among accredited residency programs during the 2020–2021 application cycle is described in Table 1. Of the 153 anesthesiology residency programs, 96 (63%) had some form of social media presence, by way of department and/or residency-affiliated account(s) on Twitter, Instagram, or Facebook. The platforms of choice for programs with social media accounts included Twitter (71, or 46%) and Instagram (67, or 44%), followed by Facebook (47, or 31%). Twenty-four (16%) programs appeared on all 3 platforms.

**Table 1:** Virtual Characteristics of Anesthesiology Residency Programs, 2020–2021

Virtual Opportunities and Social Media Platforms	n (%)
Total Anesthesiology Residency Programs	153 (100)
Programs with ≥1 Social Media Account(s)	96 (63)
Programs on Twitter, Instagram, and Facebook^*a*^	24 (16)
Virtual Open Houses	
**•** Programs with Advertised Virtual Open Houses	91 (59)
**•** Programs with Virtual Open Houses on Program Website	20 (13)
**•** Programs with Virtual Open Houses on ASA Link	82 (54)
**•** Programs with Virtual Sub-Internships on VSAS	2 (1)
**•** Programs with > 1 Advertised Virtual Open House	37 (24)
Twitter	
**•** Programs on Twitter^*a*^	71 (46)
**•** Programs with a Department Twitter	61 (40)
**•** Programs with a Residency-Affiliated Twitter	19 (12)
**•** Programs with Open House Opportunities on Twitter	38 (25)
**•** Programs with > 1 Open House Opportunity on Twitter	21 (14)
Instagram	
**•** Programs on Instagram^*a*^	67 (44)
**•** Programs with a Department Instagram	22 (14)
**•** Programs with a Residency-Affiliated Instagram	47 (31)
**•** Programs with Open House Opportunities on Instagram	33 (22)
**•** Programs with > 1 Open House Opportunity on Instagram	16 (10)
Facebook	
**•** Programs on Facebook^*a*^	47 (31)
**•** Programs with a Department Facebook	38 (25)
**•** Programs with a Residency-Affiliated Facebook	10 (6)
**•** Programs with Open House Opportunities on Facebook	13 (8)
**•** Programs with > 1 Open House Opportunity on Facebook	6 (4)

^a^Programs with department and/or residency-affiliated account(s)

**Fig. 1:**
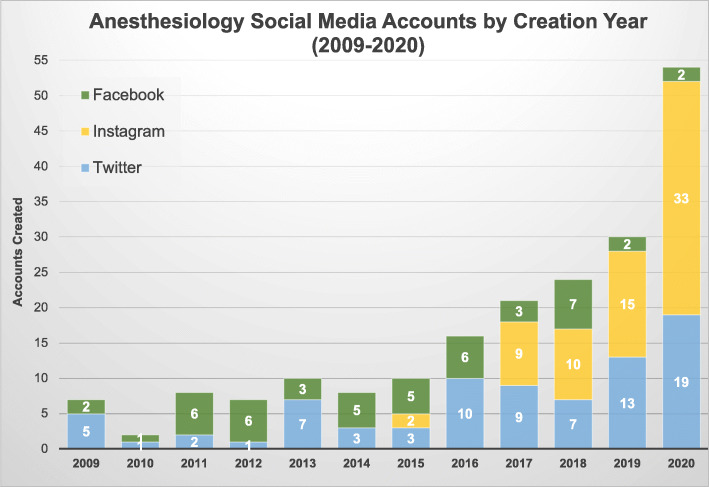
Anesthesiology Social Media Accounts by Creation Year, 2009–2020

**Fig. 2:**
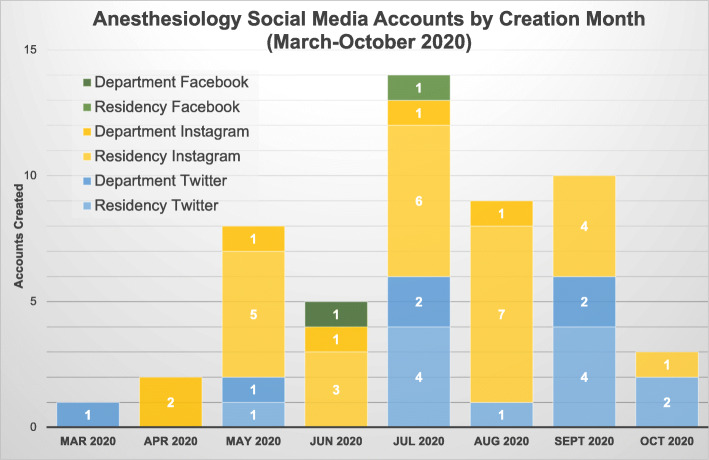
Anesthesiology Social Media Accounts by Creation Month, March–October 2020

In total, 197 social media accounts existed across all anesthesiology programs. Annual account creation steadily increased from 7 in 2009 to 30 in 2019; this growth further increased to 54 accounts in 2020 (Fig. 1). Of the 76 residency-affiliated accounts across all platforms, 40 were created after March 1, 2020 (COVID-19 onset in the United States), and 14 of the 121 department accounts were formed in this timeframe (Fig. 2). Additionally, Instagram accounts topped all new account creations for anesthesiology residency accounts (26 out of 40) after the COVID-19 pandemic onset. Department accounts generated during this time were mostly on Twitter and Instagram (both 6 out of 14).

Most anesthesiology residency programs (59%) advertised virtual open houses across social media and program and ASA-sponsored websites for prospective applicants. Moreover, Twitter (25%), Instagram (22%), and Facebook (8%) were used by programs to advertise these opportunities. More specifically, 37 (24%) programs offered multiple events across all online platforms. Furthermore, virtual open houses were listed on 20 (13%) programs’ department or residency websites. A link on the ASA website with an editable document listed 82 programs with virtual open house opportunities. Two sub-internships were offered in a virtual format on the VSAS database. No programs offered virtual sub-internship opportunities on their websites or social media platforms.

## Discussion

Anesthesiology residency programs have increasingly utilized social media in their efforts to recruit applicants over the past few years. The COVID-19 pandemic appears to have accelerated programs’ use of social media. Annual account growth has more than quadrupled in the past 5 years and nearly doubled since the pandemic onset in March 2020. Despite the increasing importance of social media in announcing virtual opportunities, fewer than half of programs have employed multiple social media platforms to reach prospective applicants. Nevertheless, Instagram and Twitter appear to be the preferred platforms for outreach.

Although expanding their social media presence demonstrates progress in virtual engagement with applicants, programs may benefit from hosting forums, e.g., open houses, where applicants can engage in live conversation with program personnel. This could enable applicants to make better informed decisions when selecting the prospective institutions they plan to apply, visit, interview and ultimately attend [9]. These opportunities are likely valuable for individuals without anesthesia residency programs at their home institutions, who would otherwise utilize away rotations to familiarize themselves with residency programs. Approximately half of all programs offered virtual open houses, with only one-fifth holding multiple events. Offering additional opportunities may seem daunting for residencies; however, these likely do not exclusively benefit applicants—it may also streamline programs’ efforts by refining their applicant pool to individuals who are best suited for their program. Moreover, virtual events may enable programs to gauge personalities and interest among applicants ahead of their interview or application review. This holds greater significance in a time when programs are expected to receive more applications than usual due to the elimination of interview travel expenses [10].

Although virtual sub-internships could also serve as opportunities for more expansive outreach, the existence of only two such programs may be attributed to the value of hands-on experience in anesthesia education. Nevertheless, programs may continue to offer videoconferences in future application cycles, as they are cost-effective, compliant with social distancing guidelines, and desired as an option by both applicants and residents alike [11]. If future application cycles preserve virtual elements, such as videoconferences and virtual open houses, it would be logical to assume that medical student applicants would take advantage of these opportunities. To do so would allow applicants access to program characteristics and other basic information that could make programs desirable for a trainee with minimal travel or time expense. While in person interviews afford applicants the opportunity to experience the area and medical center firsthand, as well as connect in person with educators and current residents, this time and travel can be prohibitive, and virtual experiences can provide an alternative to or supplement traditional in person activities.

Moving forward, it will be crucial to understand the effect of social media and virtual events on anesthesiology residency programs during the 2020–2021 application cycle. Individual programs would need to be surveyed on whether virtual tools enhanced their recruitment efforts, attracted new pools of applicants to their program, or transformed their approach to applicant outreach for upcoming interview seasons. Future investigation should explore how the landscape of anesthesiology residency applications has been altered by the emergence of virtual recruitment.

## Conclusion

The COVID-19 pandemic has posed unique challenges for the residency application process. In response, there has been exponential growth in social media accounts created by anesthesiology programs over the past year. Most programs have relied upon social media platforms to advertise virtual open houses and assist with anesthesiology resident recruitment.

## Data Availability

The datasets used and/or analyzed during the current study are available from the corresponding author on reasonable request.

## Footnotes

1 Open Houses are informational sessions about residency programs, involving live presentations and/or informal meet-and-greets between applicants and various residency program representatives, including residents, faculty, and staff.

2 Sub-internships (sub-is), also known as “away rotations” or “auditions,” are clinical rotations of fourth-year medical students which are offered by programs affiliated separately from their home institutions. These are typically in students’ specialty of interest and often utilized to demonstrate their commitment to the outside program.
